# Assisted reproductive techniques do not impact late neurodevelopmental outcomes of preterm children

**DOI:** 10.3389/fped.2023.1123183

**Published:** 2023-06-19

**Authors:** Tiphaine Lefebvre, Cyril Flamant, Marion Olivier, Géraldine Gascoin, Pierre-Emmanuel Bouet, Jean-Christophe Roze, Paul Barrière, Thomas Fréour, Jean-Baptiste Muller

**Affiliations:** ^1^ Department of Biology and Reproductive Medicine, University Hospital of Nantes, Nantes, France; ^2^ Faculty of Medicine, University of Nantes, Nantes, France; ^3^ Department of Neonatal Medicine, University Hospital of Nantes, Nantes, France; ^4^ Loire Infant Follow-up Team (LIFT) Network, Pays de La Loire, France; ^5^ Department of Neonatal Medicine, University Hospital of Angers, Angers, France; ^6^ Department of Obstetrics and Gynecology, University Hospital of Angers, Angers, France; ^7^ Center for Research in Transplantation and Immunology, Inserm, University of Nantes, Nantes, France

**Keywords:** assisted reproductive technology, *in vitro* fertilization, prematurity, cognitive development, follow-up

## Abstract

**Objective:**

Assisted reproductive technology (ART) increases the rate of preterm births, though few studies have analyzed outcomes for these infants. No data are available on 4-year-old children born prematurely after ART. The objective was to investigate whether ART affect the neurodevelopmental outcomes at 4 years in preterm infants born before 34 weeks of gestational age (GA).

**Methods and results:**

A total of 166 ART and 679 naturally conceived preterm infants born before 34 weeks GA between 2013 and 2015 enrolled in the Loire Infant Follow-up Team were included. Neurodevelopment was assessed at 4 years using the age and stage questionnaire (ASQ) and the need for therapy services. The association between the socio-economic and perinatal characteristics and non-optimal neurodevelopment at 4 years was estimated. After adjustment, the ART preterm group remained significantly associated with a lower risk of having at least two domains in difficulty at ASQ: adjusted odds ratio (aOR) 0.34, 95% confidence interval (CI) (0.13–0.88), *p* = 0.027. The factors independently associated with non-optimal neurodevelopment at 4 years were male gender, low socio-economic level, and 25–30 weeks of GA at birth. The need for therapy services was similar between groups (*p* = 0.079). The long-term neurodevelopmental outcomes of preterm children born after ART are very similar, or even better than that of the spontaneously conceived children.

## Introduction

It is estimated that 2%–6% of all births are conceived through assisted reproductive technology (ART) (1). Although reproductive medicine techniques have been used for more than 40 years and are generally considered to be safe, some studies nevertheless suggested a slight increase in congenital comorbidities, pregnancy complications, and neonatal morbidity, as well as an increase in epigenetic abnormalities in children born through *in vitro* fertilization (IVF) with poorly understood etiological mechanisms (2).

Prematurity, defined by live birth before 37 weeks of gestational age (GA), is a major and growing health issue of concern. It is associated with an increased risk of congenital malformation, mortality and neonatal morbidities, and long-term complications such as neurodevelopmental sequelae. In 2010, prematurity concerned 15 million births worldwide (11% of the total birth) (3). The increasing rate of prematurity has been partly linked to the increased use of ART worldwide (4). Indeed, several studies, including some meta-analyses, reported an increased risk of prematurity and low birth weight after ART, regardless of multiple pregnancy status (5).

Some studies analyzed the neurodevelopmental outcomes in children conceived through ART. Their results were compiled and summarized in systematic reviews, which concluded to the absence of significant difference in the neurodevelopmental wellbeing between children born after ART and children who are naturally conceived (2). Although motor and cognitive development does not appear to be affected by the method of conception, the parental educational level and maternal age might be confounding factors, as they play a key role in cognitive development (2, 6). Available scientific data are therefore mostly reassuring.

Relatively few studies have specifically analyzed neonatal outcomes in preterm infants born after ART, without reporting significant differences with the naturally conceived ones (7–9). Interestingly, only four studies compared the neurodevelopmental outcome of preterm infants born after ART with preterm infants who were spontaneously conceived (10–13). Abdel-Latif et al. focused on extreme prematurity (<29 weeks GA) and on functional disability at 2–3 years defined by developmental delay, cerebral palsy, or sensorial impairment. ART conceptions were associated with adverse neurodevelopmental outcome only among high-risk infants born at 22–26 weeks GA (10). Molines et al. reported a lower probability of non-optimal neurodevelopment at 2 years [using the age and stage questionnaire (ASQ) or development quotient of Brunet–Lezine test] after ART than after spontaneous pregnancy in a cohort of preterm children born before 34 weeks GA (11). The third one concluded to a similar neurodevelopmental outcome at 24–36 months of age, using Gross Motor Function Classification System and Denver II, in 125 preterm infants born between 24 and 36 weeks GA either after ART or after spontaneous multiple pregnancy (12). Recently, Roychoudhury et al. reported a lower risk of neurodevelopmental impairment at 18–24 months of preterm infants born before 29 weeks GA after ART. They also emphasized the importance of carrying out a long-term follow-up study to explore learning disabilities and cognitive impairment (13).

Among the tools that explore general child development, the ASQ is a widely recommended general development assessment tool, gathering the observation of the parents of child skills (14). Furthermore, ASQ is currently being used in large national cohorts of preterm infants as a screening tool for developmental delays, as well as in large longitudinal studies of children born through ART such as the Upstate KIDS study (15). It is composed of an age-specific questionnaire, from 4 to 60 months. To date and to the best of our knowledge, no data are available in the literature on neurodevelopment or the need for referral to therapy services at age 4 for children born prematurely after ART. Comparing such data with those obtained in the spontaneously conceived preterm children would be relevant to help health professionals provide reliable information to parents.

The main objective of this study was to evaluate the impact of ART on the neurodevelopmental outcomes at 4 years in preterm infants born before 34 weeks GA compared with preterm infants who were spontaneously conceived. The secondary objective was to assess the need for therapy services at the same age.

## Materials and methods

### Population

All infants born alive before 34 weeks GA between January 2013 and September 2015 and enrolled in the Loire Infant Follow-up Team (LIFT) were included. This regional network includes 24 maternity hospitals, with 3 neonatal intensive care units and 6 ART centers. Infants with congenital malformation were excluded from the analysis. Infant whose parents declined their participation in the LIFT follow-up program were also excluded (16).

The following pregnancy-related maternal and neonatal data were prospectively collected: mode of conception and the technique used if it was an assisted conception [ovarian stimulation with or without intrauterine insemination, IVF with or without intra-cytoplasmic sperm injection (ICSI)], socio-economic status (SES) (INSEE classification, www.insee.fr/fr/information/2406153), universal health coverage and maternal age, gender, birth weight [expressed in relation to GA as *z*-scores for standard deviations (SD)] from Olsen growth curves, gestational age, and neonatal morbidities.

### Outcome

Neurodevelopment at 4 years old was assessed using the ASQ in addition to medical consultation. The ASQ is a parent-completed screening test composed of 21 age-specific questions covering the age range of 4–60 months (17). In the present study, the 48-month questionnaire (second version) was used. It includes 30 developmental items divided into five domains (six items each) of child capacities: communication abilities, gross motor skills, fine motor skills, problem solving abilities, and personal-social skills. For each item, three responses are possible, depending on whether the child performs the task: “Yes” (10 points), “Sometimes” (5 points), and “Not Yet” (0 point). The total score for each domain is obtained by adding the scores of the six items. The overall ASQ score is established by combining the scores for the five domains, with a maximum global ASQ score of 300 points. Parents completed the ASQ between 1 month before and 1 month after the 48-month target age. The mean ASQ score as well as the difficulty in at least one or two domains at ASQ were reported. The need for therapy services at 4 years of age was defined as one or more appointments with a speech, physical, or occupational therapist or an orthoptist, or as support in a child development center (16).

### Statistical analysis

Quantitative variables are presented as mean ± SD, and qualitative variables are presented as numbers of subjects (*n*) and percentages (%). The differences were analyzed with a *χ*^2^ test or Fisher exact test for discrete variables with expected values of less than 5. Student’s *t*-test and the non-parametric Mann–Whitney *U* test were used to compare continuous variables.

First, the study sample population of preterm infants with a 4-year ASQ assessment was compared with the lost to follow-up population in LIFT cohort to study possible selection biases. Then, the characteristics of ART preterm group were compared with the naturally conceived preterm group. Second, the mean ASQ score and difficulty in at least one or two domains at ASQ were compared between ART and spontaneous preterm groups. The respective association between the socio-economic, pregnancy, and neonatal characteristics and non-optimal neurodevelopment at 4 years was estimated in bivariate analysis. Two regression logistic models including ART variable as a main factor was computed to define adjusting factors that could influence difficulty in at least in one or two domains. As a sensitivity analysis, we fitted a generalized estimating equation (GEE) model to account for within-subject correlation due to the multiple pregnancy rate, and check our regression logistic model results. The GEE model used the mother level as a cluster level, and the same variables included in the initial regression logistic model. Finally, a missing data imputation was realized to verify that the results of multivariate analysis in the whole population of preterm children with visit at 4 years were not biased compared with the study sample of children with complete ASQ at 4 years.

A statistical significance was defined at *p*-value less than .05 for the whole analysis. All of the statistical analyses were performed using R software (version 3.6.2).

## Results

### Study population

**Figure 1:**
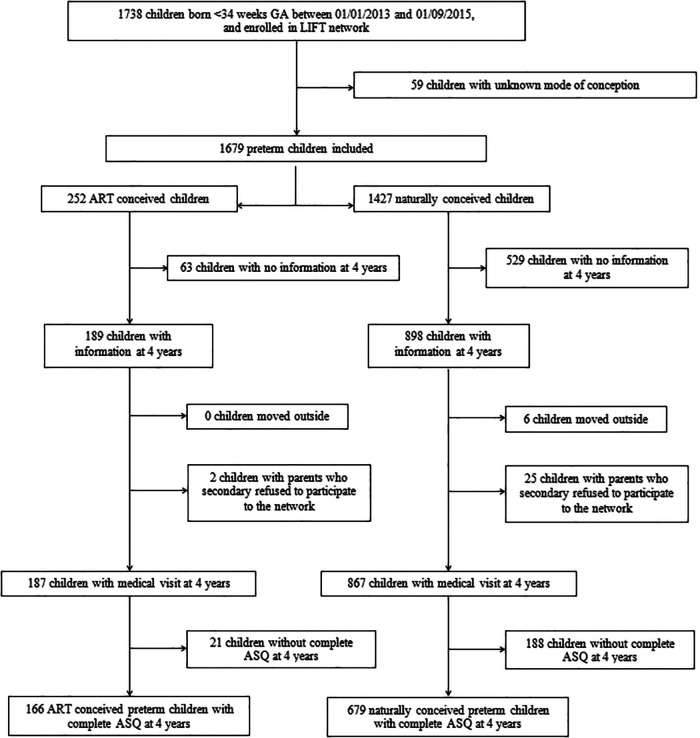
Flowchart diagram.

A total of 845 of the 1,679 preterm children enrolled in the regional LIFT cohort with a known mode of conception status were assessed at 4 years old using the ASQ (50.3%). Among them, 166 were born after ART (19.6%) and 679 after spontaneous pregnancy (80.4%) (shown in Figure 1).

The neonatal and maternal characteristics, as well as the socio-economic status (SES) and ASQ at 4 years, in both naturally conceived and ART preterm children are presented in Table 1. The proportion of multiple pregnancy, mean maternal age, and socio-economic status were significantly higher in ART preterm children than in the naturally conceived ones (Table 1).

**Table 1:** Comparison of neonatal characteristics, maternal characteristics, and socio-economic status.

	Naturally conceived preterm children (*n* = 679)	ART-conceived preterm children (*n* = 166)	*p*-value
Neonatal characteristics			
Male gender (%)	353 (52.0)	82 (49.4)	0.61
Weeks GA	30.7 ± 2.28	30.98 ± 2.07	0.16
Weeks GA, class (%)			0.19
25–27	82 (12.1)	12 (7.2)	
28–31	285 (42)	71 (42.8)	
32–34	311 (45.9)	83 (50)	
Birth weight			0.58
Weight (g)	1,487.7 ± 450.5	1,466.17 ± 373.5	
< −1 *z*-score (%)	165 (24.3)	47 (28.3)	0.318
Maternal characteristics			
Multiple pregnancy (%)	190 (28.1)	118 (71.1)	<0.001
ART (%)			
IVF ± ICSI		96 (57.8)	
Ovarian stimulation		31 (18.7)	
Intrauterine insemination		15 (9)	
Oocyte donation		24 (14.5)	
Maternal hypertension	116 (17.1)	32 (19.3)	0.581
Threatening premature birth	319 (47)	66 (39.8)	0.112
Chorioamnionitis	21 (3.1)	5 (3.0)	1.00
Maternal age (years)	30.2 ± 5.1	33.8 ± 5.6	<0.001
Maternal age (%)			<0.001
16–24 years	72 (14)	7 (4.8)	
25–30 years	205 (39.9)	40 (27.2)	
31–35 years	150 (29.2)	46 (31.3)	
>35 years	87 (16.9)	54 (36.7)	
Socio-economic status			
Sibling	2.2 ± 1.0	2.1 ± 0.7	0.60
SES			0.031
Low SES (%)	55 (9.3)	6 (3.8)	
High SES (%)	128 (18.9)	47 (28.3)	0.01

ART, assisted reproductive technology; GA, gestational age; IVF, *in vitro* fertilization; ICSI, intra-cytoplasmic sperm injection; SES, socio-economic status.

Results are expressed as mean ± SD and number (%).

*p* < 0.05: significant.

The respective neonatal and maternal characteristics of the children included in the study (*n* = 845) and those theoretically eligible but excluded because of a lack of information at 4 years (*n* = 592) are presented in Supplementary Table S1. The proportion of ART-conceived children was significantly higher in children with visit at 4 years than in children without visit at 4 years. The gestational age at birth, multiple pregnancy rate, high socio-economic status, and maternal age at birth were significantly higher in children included in the study than in those excluded.

### Four-year neurodevelopmental outcomes

**Figure 2:**
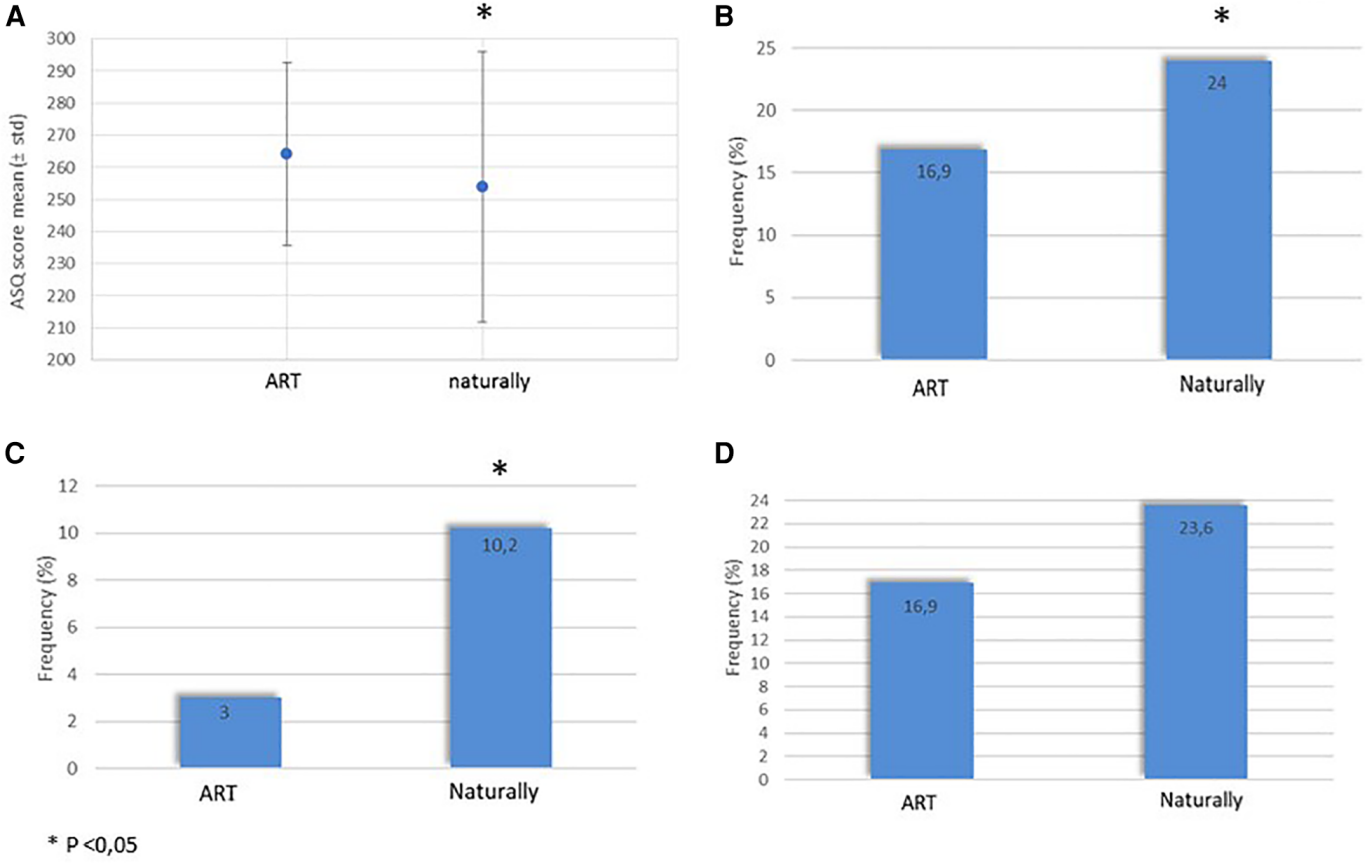
Neurodevelopment at 4 years in ART and naturally conceived preterm. (**A**) Mean ASQ score. (**B**) Difficulty in at least one ASQ domain. (**C**) Difficulty in at least two ASQ domains. (**D)** Need for therapy services at 4 years. ART, assisted reproductive technology; ASQ, age and stage questionnaire.

The ASQ neurodevelopment assessment at 4 years is presented in Figure 2. The mean ASQ score was significantly higher (shown in Figure 2A) and difficulty in at least one or two domains were significantly lower in ART preterm group than in the naturally conceived preterm children (shown in Figures 2B, C). The need for therapy services was not significantly different between both groups (shown in Figure 2D).

Maternal, neonatal, and socio-economic characteristics associated with neurodevelopment are presented in Table 2. After adjustment in logistic regression model, including male gender, weeks gestational age, birth weight *z*-score, ART, multiple pregnancy rate, SES, and maternal age, ART conception remained significantly associated with a lower risk of difficulty in at least two domains at ASQ in preterm infants: adjusted odds ratio (aOR) 0.34, 95% CI (0.13, 0.88), *p* = 0.028. In this model, the other factors associated with non-optimal neurodevelopment at 4 years were male gender [aOR 2.07, 95% CI (1.17, 3.65), *p* = 0.011], low socio-economic level [aOR 4.13, 95% CI (2.11, 8.07), *p* < 0.001], and gestational age at birth 25–31weeks GA [aOR 3.06, 95% CI (1.64, 5.71), *p* < 0.001]. After adjustment, difficulty in at least one domain at ASQ was not different between groups [aOR 0.87, 95% CI (0.54–1.41), *p* = 0.58]. The adjusted GEE model confirmed our results, not modifying noticeably the odds ratios for the risk of difficulty in two domains at ASQ, for ART [aOR 0.37, 95% CI (0.15, 0.94), *p* = 0.037] and for other factors (male gender: aOR 1.89, *p* = 0.022; low socio-economic level: aOR 4.06, *p* < 0.001; and gestational age at birth 25–31 weeks GA: aOR 3.05, *p* < 0.001). The correlation parameter was estimated to 0.27.

**Table 2:** Neonatal characteristics, maternal characteristics, and socio-economic status according to neurodevelopmental status at 4 years.

	ASQ no domain in difficulty at 4 years *n* = 654	ASQ ≥1 domain in difficulty at 4 years *n* = 191	*p*-value	ASQ <2 domains in difficulty at 4 years *n* = 771	ASQ ≥2 domains in difficulty at 4 years *n* = 74	*p*-value
Neonatal characteristics						
Male gender (%)	311 (47.6)	124 (64.9)	<0.001	387 (50.2)	48 (64.9)	0.022
Weeks GA	30.9 ± 2.2	30.3 ± 2.5	<0.001	30.9 ± 2.2	29.8 ± 2.5	<0.001
Weeks GA class (%)			0.016			0.001
25–27	65 (9.9)	29 (15.3)		80 (10.4)	14 (19.2)	
28–31	268 (41)	88 (46.3)		316 (41)	40 (54.8)	
32–34	321 (49.1)	73 (38.4)		375 (48.6)	19 (26)	
Birth weight						
Weight (g)	1 506.9 ± 408.5	1 399.7 ± 513.1	0.003	1 495.9 ± 424.7	1 344.6 ± 526.2	0.004
< −1 *z*-score (%)	147 (22.5)	65 (34)	0.002	186 (24.1)	26 (35.1)	0.052
Maternal characteristics						
Multiple pregnancy (%)	262 (40.2)	46 (24.1)	<0.001	292 (38)	16 (21.6)	0.008
ART conception (%)	138 (21.1)	28 (14.7)	0.062	161 (20.9)	5 (6.8)	0.002
Maternal age (years)	31.2 ± 5.3	30.3 ± 5.9	0.057	31.2 ± 5.4	29.3 ± 5.7	0.013
Maternal age (%)			0.011			0.018
16–24 years	49 (9.7)	30 (19.4)		65 (10.8)	14 (23)	
25–30 years	191 (37.7)	54 (34.8)		220 (36.7)	25 (41)	
31–35 years	152 (30.0)	44 (28.4)		183 (30.5)	13 (21.3)	
>35 years	114 (22.5)	27 (17.4)		132 (22)	9 (14.8)	
Socio-economic status						
Siblings	2.2 ± 0.9	2.1 ± 1.1	0.71	2.2 ± 0.9	2.2 ± 1.2	0.63
SES						
Low SES (%)	37 (6.3)	24 (14.7)	0.001	45 (6.6)	16 (25)	<0.001
High SES (%)	148 (22.6)	27 (14.1)	0.014	167 (21.7)	8 (10.8)	0.04

ART, assisted reproductive technology; GA, gestational age; SES, socio-economic status.

Results are expressed as mean ± SD and number (%).

*p* < 0.05: significant.

### Missing data imputation analysis

The results of the logistic regression model obtained after imputation analysis in the overall cohort of 1,054 children with a visit at 4 years were not significantly different from those obtained in the study sample of 845 children with complete ASQ at 4 years. After adjustment, ART conception remained significantly associated with a lower risk of difficulty in at least two domains at ASQ. Male gender, low SES, and gestational age 25–30 weeks were also independently and significantly associated with a higher risk of non-optimal neurodevelopment at 4 years.

## Discussion/conclusion

In this study conducted in a large cohort of preterm children born before 34 weeks GA, the neurodevelopmental outcomes at 4 years were not found to be negatively impacted by ART conception. Actually, the mean ASQ score at 4 years was even significantly higher, and the frequency of non-optimal neurodevelopment illustrated by a difficulty in at least two domains was significantly reduced in ART-conceived preterm children compared with the naturally conceived ones. ART-conceived preterm children also did not require significantly more therapy services than the naturally conceived ones.

As widely described in the field of ART, we also reported a significant increase in the rate of multiple pregnancies and a higher maternal age in ART-conceived children as compared with natural conceptions (18). Previously, Farhi et al. already published similar long-term cognitive achievement outcome at school age between children born at term after ART and those spontaneously conceived. Our results are consistent with well-known neonatal characteristics of preterm children associated with poor neurodevelopmental outcomes (20), such as earlier weeks GA at birth, lower socio-economic status, and male gender (birth weight < −1 *z*-score is significant for ASQ ≥1 domain in difficulty). Our results obtained at 4 years confirm and reinforce those previously reported in preterm children evaluated at 2 years (10–12), showing that preterm children conceived by ART have reduced risk of non-optimal neurodevelopment as compared with the naturally conceived ones. Consistently with the literature, we found that women who underwent ART had a higher SES, which is a protective factor against neurodevelopmental disorders (21). However, we confirmed that preterm children born after ART still had fewer neurodevelopmental disorders than those conceived naturally, even after adjusting for SES, thus highlighting the specificity of ART-conceived preterm children. This is also illustrated in our study by the lower (although not significant) referral to therapy services in the ART preterm group, while the literature reported that women with high SES were more likely to refer children to therapy services (22).

Long-term developmental outcomes in preterm twins vs. singletons have been extensively discussed in the literature before (23). In addition, there is a risk of neurodevelopmental disorders associated with monochorionic pregnancies; in our cohort, the number of children born from a monochorionic pregnancy is not different in the ART vs. the naturally conceived group (11.9% vs. 11.4%, respectively). Higher maternal age has been suggested to stimulate infant neurodevelopment (24). These two factors have therefore been considered here as confounding factors. After adjustment in multivariate analysis, we observed that ART conception remained independently associated with a reduction in the risk of non-optimal neurodevelopment. Other factors stemming from the mother may be implicated in preterm neurodevelopmental outcomes. Parental factors that support neurodevelopment in ART situations are complex to explain. Nevertheless, sending of positive signals and repeated interaction attempts have been reported to be specific characteristics of the behavior of ART mothers (25). It can thus be speculated that these better mother–baby interactions support and enhance child neurodevelopment (26).

The main strength of this study is the large number of preterm children monitored at 4 years. Multicentric inclusion also allowed a short inclusion period, ensuring consistency of care. Multivariate analysis that allowed controlling for main confounding factors, missing data imputation analysis, and GEE modeling reinforced the results. Finally, ASQ is a well-known and validated tool, especially in preterm children (17). Nevertheless, the continuous ASQ score as a cognitive predictive tool have a limited specificity and should be used with caution. As proposed by Sices et al., using the criteria of difficulty in at least one or two domains of development enhanced specificity (96% for difficulty in at least two domains vs. 84% for difficulty in at least one domain) (27).

We also acknowledge that our study has some limitations. The comparison of study and lost to follow-up populations showed that ART-conceived preterm children were more closely monitored than the spontaneously conceived ones, probably because of the higher socio-economic status and parental investment in this group. However, this limitation was partly overcome by missing data imputation.

In conclusion, long-term neurodevelopment of preterm children born after ART is very similar, or even better than that of the spontaneously conceived children. This is reassuring, both for infertility specialists and for parents who conceived through ART in case of threatened preterm birth.

## Data Availability

The raw data supporting the conclusions of this article will be made available by the authors, without undue reservation.
