# Supplementary material for: Assisted reproductive techniques do not impact late neurodevelopmental outcomes of preterm children
Source: Front Pediatr. 2023 Jun 19;11:1123183. doi: 10.3389/fped.2023.1123183 (PMC10315460; doi:10.3389/fped.2023.1123183)
Supplement: Supplementary file 1 [file Table1.docx]

**Supplemental Table 1. Comparison of neonatal and maternal characteristics of preterm children included in the study and preterm children eligible but excluded because of a lack of visit at 4 years of age.**

|  | **Visit at 4**  **(n=845)** | **No visit at 4**  **(n=592)** | ***p* value** |
| --- | --- | --- | --- |
| **Neonatal characteristics** |  |  |  |
| ART conception (%) | 166 (19.6) | 63 (11.1) | <0.001 |
| Male gender (%) | 435 (51.5) | 316 (55.4) | 0.16 |
| Weeks GA, mean+/-SD | 30.8 +/- 2.3 | 30.5 +/- 2.8 | 0.03 |
| Birth weight  Weight g, mean (SD)  < -1 z score (%) | 1482.7+/- 436.3  212 (25.1) | 1439.3+/-486.6  167 (29.3) | 0.08  0.087 |
| **Maternal characteristics** |  |  |  |
| Multiple pregnancy (%) | 308 (36.5) | 173 (30.8) | 0.032 |
| Maternal age (years), mean+/-SD | 31.0 +/- 5.4 | 29.6 +/- 5.8 | <0.001 |
| Socio-economic status  Low SES (%)  High SES (%) | 61 (8.2)  175 (20.7) | 62 (24.5)  49 (8.6) | <0.001  <0.001 |

*Results are expressed as mean +/- standard deviation and number (percentage)*

*p< 0.05: significant*

*ART: Assisted Reproductive Technology; GA: Gestational Age; IVF: in vitro Fertilization; ICSI: Intra-cytoplasmic Sperm Injection; SES: Socio-economic status*
